# ChatGPT in Answering Queries Related to Lifestyle-Related Diseases and Disorders

**DOI:** 10.7759/cureus.48296

**Published:** 2023-11-05

**Authors:** Himel Mondal, Ipsita Dash, Shaikat Mondal, Joshil Kumar Behera

**Affiliations:** 1 Physiology, All India Institute of Medical Sciences, Deoghar, IND; 2 Biochemistry, Saheed Laxman Nayak Medical College and Hospital, Koraput, IND; 3 Physiology, Raiganj Government Medical College and Hospital, Raiganj, IND; 4 Physiology, Nagaland Institute of Medical Science and Research, Kohima, IND

**Keywords:** public health education, patient, internet, non-communicable disease, healthcare, language model, lifestyle, education, chatgpt, artificial intelligence

## Abstract

Background

Lifestyle-related diseases and disorders have become a significant global health burden. However, the majority of the population ignores or do not consult doctors for such disease or disorders. Artificial intelligence (AI)-based large language model (LLM) like ChatGPT (GPT3.5) is capable of generating customized queries of a user. Hence, it can act as a virtual telehealth agent. Its capability to answer lifestyle-related diseases or disorders has not been explored.

Objective

This study aimed to evaluate the effectiveness of ChatGPT, an LLM, in providing answers to queries related to lifestyle-related diseases or disorders.

Methods

A set of 20 lifestyle-related disease or disorder cases covering a wide range of topics such as obesity, diabetes, cardiovascular health, and mental health were prepared with four questions. The case and questions were presented to ChatGPT and asked for the answers to those questions. Two physicians rated the content on a three-point Likert-like scale ranging from accurate (2), partially accurate (1), and inaccurate (0). Further, the content was rated as adequate (2), inadequate (1), and misguiding (0) for testing the applicability of the guides for patients. The readability of the text was analyzed by the Flesch-Kincaid Ease Score (FKES).

Results

Among 20 cases, the average score of accuracy was 1.83±0.37 and guidance was 1.9±0.21. Both the scores were higher than the hypothetical median of 1.5 (p=0.004 and p<0.0001, respectively). ChatGPT answered the questions with a natural tone in 11 cases and nine with a positive tone. The text was understandable for college graduates with a mean FKES of 27.8±5.74.

Conclusion

The analysis of content accuracy revealed that ChatGPT provided reasonably accurate information in the majority of the cases, successfully addressing queries related to lifestyle-related diseases or disorders. Hence, initial guidance can be obtained by patients when they get little time to consult a doctor or wait for an appointment to consult a doctor for suggestions about their condition.

## Introduction

Lifestyle-related diseases or disorders, often referred to as non-communicable diseases (NCDs), have become a significant global health concern [1]. These diseases, including conditions like obesity, diabetes, cardiovascular diseases, mental health disorders, and certain types of cancers, are primarily influenced by modifiable lifestyle factors such as unhealthy diet, lack of physical activity, tobacco use, and excessive alcohol consumption [2,3].

As majority of the lifestyle-related diseases or disorders may not cause immediate ill health to the extent of seeking medical help, people ignore it a lot [4]. However, when individuals seek information and guidance to better understand these lifestyle-related diseases and make informed decisions about their health, the availability of accurate and accessible information becomes crucial [5].

The advancements in artificial intelligence (AI) and natural language processing have paved the way for large language models (LLMs) like ChatGPT, which can assist in providing answers to a wide range of queries related to health and diseases. LLMs like ChatGPT may help in addressing patients' health-related queries. It can provide quick access to general health information, offering explanations about common symptoms, medical conditions, and treatment options. It has been tested in various domains like medical diagnostics; education; telemedicine; and customized content generation for information, education, and communication purposes [6-8]. Along with its advantages, there are limitations to consider. ChatGPT is not a substitute for professional medical advice, diagnosis, or treatment, and its responses are based on general knowledge up to a specific date. It cannot access real-time patient data or conduct physical examinations, making it unsuitable for diagnosing specific medical conditions. Additionally, the model can occasionally provide inaccurate or outdated information [9]. Hence, the effectiveness and reliability of these models in addressing queries specific to lifestyle-related diseases or disorders require careful evaluation.

This study aimed to analyze the content and readability of ChatGPT's responses when answering queries related to lifestyle-related diseases or disorders. The findings of this study will contribute to our understanding of the strengths and limitations of ChatGPT in addressing queries related to lifestyle-related diseases or disorders.

## Materials and methods

Type, settings, and ethics

This is a cross-sectional data audit from online resources where the data does not originate from any animal or human research participants. The study was conducted in July 2023 with a personal computer connected to the World Wide Web with the help of a personal broadband connection. No institutional resources were used for this study. Data generated by the language model was not published in the manuscript. Hence, this study does not require human or animal research committee review.

Case vignettes

**Figure 1:**
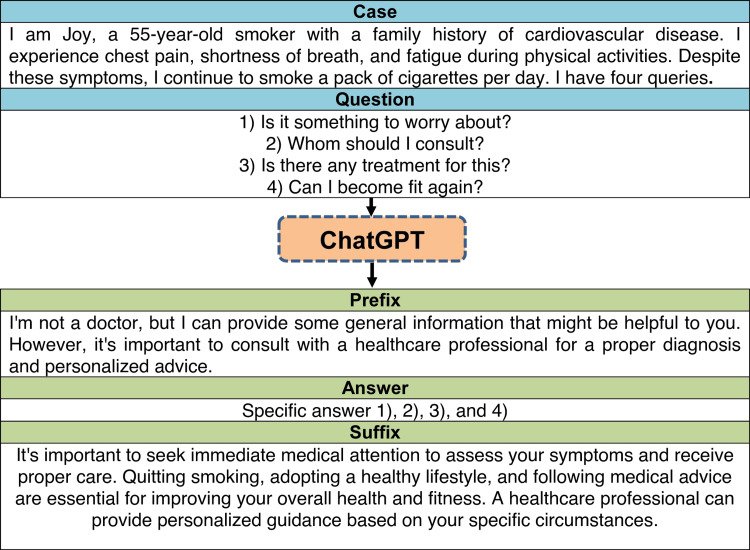
The case, question, and reply pattern generated by ChatGPT

In this study, we utilized a sample of 20 cases mimicking individuals seeking information related to lifestyle-related diseases or disorders. These cases were created for this study after considering the common lifestyle-related diseases and disorders to simulate common questions that individuals may have in real-world scenarios. The content validity was checked by an academic public health expert. Each case consisted of a specific query related to a particular aspect of lifestyle-related diseases. There were four questions followed by each case presentation. The presentation was to apprise ChatGPT that the patient is seeking information from the LLM. A sample question is available in Figure 1.

Tools

Artificial Intelligence

The cases were presented to ChatGPT (https://chat.openai.com) (GPT3.5, May 24, 2023 version), an LLM, and the responses generated by ChatGPT were collected and stored for further analysis. ChatGPT responds to human queries by leveraging its pre-trained language model, which has been trained on a diverse range of internet text. It generates responses based on patterns, context, and knowledge acquired during training. However, the version we used in this study did not have real-time access to external information and was limited to the knowledge it has been trained on, which cuts off in September 2021 [9].

Sentiment analysis

The sentiment of the text was analyzed by a free basic version of the program MonkeyLearn available at: https://monkeylearn.com/sentiment-analysis-online. This program analyzes the text and provides the result according to the analysis free of cost. Sentiment analysis of text is a computational technique used to determine the emotional tone or sentiment expressed in written content. It involves analyzing words, phrases, and context to classify text as positive, negative, or neutral [10].

Readability

The Flesch Reading Ease Score and Flesch-Kincaid Grade Level were obtained from a free online calculator available at: https://goodcalculators.com/flesch-kincaid-calculator. The Flesch Reading Ease Score is a measure that falls within a range of 0 to 100. A score near 100 suggests that the text is relatively easy to read, while a score close to 0 indicates that it is extremely complex and challenging to understand. For instance, a score of 70 to 80 corresponds to a text suitable for approximately a seven-grade reading level, indicating that most adults should find it quite easy to read and comprehend [11].

The Flesch-Kincaid Grade Level is a metric that indicates the educational level needed to comprehend a particular piece of writing. The Flesch-Kincaid Grade Level scores align with the grade levels of education in the United States that readers would need to have completed to understand a specific text. For instance, if a text has a Flesch-Kincaid Grade Level of 9, it implies that the reader should have completed approximately nine years of education (around ninth grade) to easily grasp the content of the document.

Content evaluation

The text was manually analyzed by two academic primary care physicians with more than five years' experience and they individually rated the answers to those four questions. There were two categories of scoring - one for accuracy and one for guidance. The qualitative rating was converted to numerical values by the following values: accurate = 2, partially accurate = 1, inaccurate = 0, adequate = 2, inadequate = 1, and misguiding = 0 for testing the accuracy and completeness of guidance, respectively.

Statistical analysis

The data were presented in terms of number, mean, and standard deviation. The intraclass correlation coefficient was used to find agreement between two raters. A one-sample median test was used to compare the score of reading score with the desired hypothetical value. For the statistical test, IBM SPSS Statistics 20 was used for all tests, and a p-value of <0.05 was considered statistically significant.

## Results

**Figure 2:**
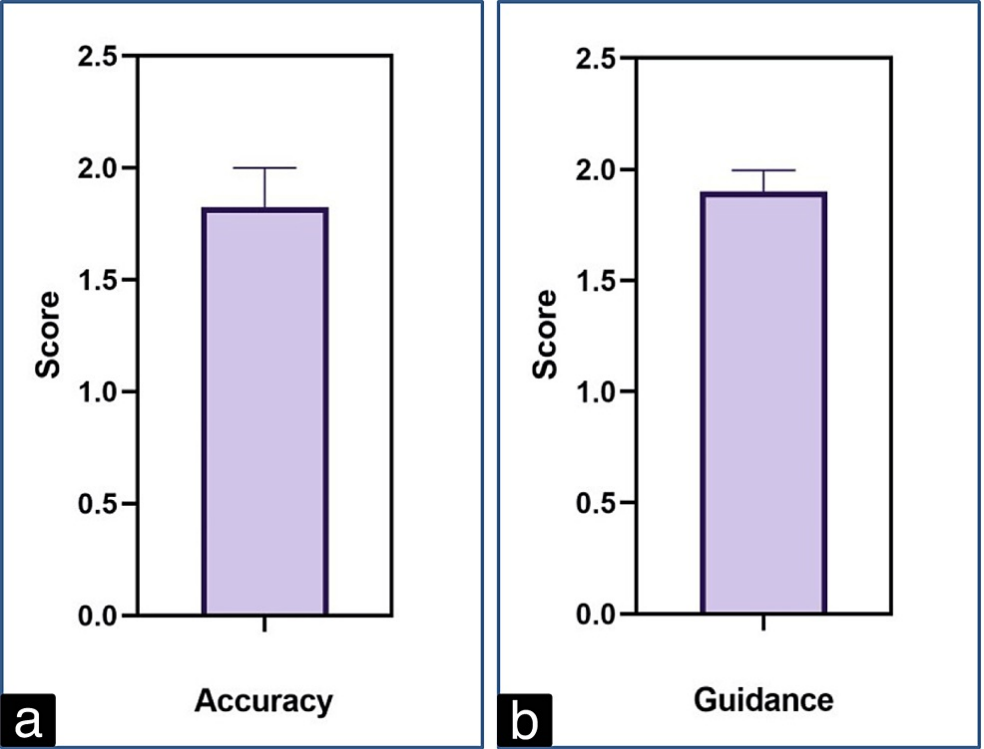
Average score of accuracy (a) and guidance (b) provided by ChatGPT

The average accuracy score was 1.83±0.37, and the guidance score was 1.9±0.21. The scores were compared statistically and found to be higher than the hypothetical median of 1.5 (Figure 2a and Figure 2b).

Two physicians demonstrated a high level of agreement for accuracy assessments, indicating consistent and reliable results. However, guidance assessments did not show significant agreement, suggesting a discrepancy in their evaluations in this aspect (Table 1).

**Table 1:** Accuracy and guidance score by two physicians and average scores *Statistically significant p-value ICC, intraclass correlation coefficient; CI, confidence interval

Parameter	Physician 1	Physician 2	Average	ICC (95% CI), p-value
Accuracy	1.85±0.37	1.8±0.41	1.83±0.37	0.91 (0.77 to 0.96), <0.0001*
Guidance	1.95±0.22	1.85±0.37	1.9±0.21	-0.18 (-2 to 0.53), 0.64

The text has an average Flesh-Kincaid Grade Level of 14.37±0.85, implying that it requires a reading comprehension level equivalent to that of a high school or college goer. The Flesch Reading Ease Score averages 27.8±5.74, indicating that the text's readability is relatively challenging. On average, each sentence consists of 20.1±1.47 words, while each word comprises 1.88±0.07 syllables. The text contains an average of 14.9±0.91 sentences and 298.9±21.74 words in total (Table 2).

**Table 2:** Characteristics of text generated in response to questions asked along with the case of lifestyle-related disease or disorder SD, standard deviation

Parameter	Mean±SD
Flesh-Kincaid Grade Level	14.37±0.85
Flesch Reading Ease Score	27.8±5.74
Average Words per Sentence	20.1±1.47
Average Syllables per Word	1.88±0.07
Sentences	14.9±0.91
Words	298.9±21.74

ChatGPT was able to provide answers with a natural (11 out of 20) and positive (9 out of 20) tone. None of the answers contained a negative tone.

## Discussion

ChatGPT's performance in providing information and guidance is generally reliable and exceeds the expected level. Since the scores are higher than the median, it indicates that ChatGPT's responses tend to be accurate and helpful in most cases, making it a valuable tool for patients seeking information and guidance. However, it is important to note that while ChatGPT's performance seems promising, it is essential to maintain caution when considering its responses as the sole source of medical advice [12,13]. The use of LLMs like ChatGPT can provide valuable insights and information, but they should complement, not replace professional medical consultation. The guidance from ChaGPT would be especially helpful when people are reluctant to get help from a healthcare professional or in a long waiting period to consult a doctor.

The high level of agreement observed between the two physicians in accuracy assessments can be attributed to the clear and objective criteria used for evaluation to reach a consensus. However, the lack of significant agreement in guidance assessments can be attributed to the more subjective nature of the evaluation process. Varied clinical experiences and decision-making styles may also contribute to the discrepancy in their evaluations.

The text generated in response to answers to the questions contained moderately long sentences and longer words, contributing to its complexity. The Flesch Reading Ease Score suggests that a college student can understand the text with ease. However, when the content is not understood, further simplification may be requested by a subsequent prompt to ChatGPT [14]. Prompt engineering is a crucial technique used to influence the responses generated by LLMs like ChatGPT. It involves crafting specific instructions or queries to elicit the desired information or tone in the model's response. A guide for prompt engineering can be accessed at https://learnprompting.org/docs/intro.

The underlying reason for ChatGPT's ability to provide answers with a natural and positive tone may lie in its training and language modeling processes. Although it can be modified by prompt engineering, common people may not engage in such engineering and ask their questions without any desired tone. During its training, ChatGPT might be exposed to a vast dataset of diverse texts, including positive and neutral language from various sources. Additionally, the AI model is designed to prioritize user satisfaction and maintain a positive conversational experience. Hence, our study suggests that ChatGPT can create a friendly and engaging interaction with a patient seeking information about their health-related issues. Hence, their tone may be better than humans and may not provide stigmatizing responses [15]. The absence of negative tones also contributes to maintaining a respectful and supportive atmosphere, which is essential when dealing with sensitive topics or providing assistance to users seeking guidance or information.

It is important to note that this study has limitations. The sample size was small, consisting of only 20 cases, which may not fully represent the range of queries and potential variations in responses. Additionally, the evaluation of content was conducted by only two human assessors, and their subjective judgment may introduce some degree of bias. Nevertheless, this study serves as an initial exploration of ChatGPT's performance in addressing common questions related to lifestyle-related diseases or disorders, providing valuable insights for further research and improvement in the field of AI-driven healthcare information provision.

## Conclusions

This study evaluated the effectiveness of ChatGPT, an LLM, in providing answers to queries about lifestyle-related diseases or disorders. Overall, ChatGPT showed promising results, demonstrating accuracy in its responses and providing adequate guidance for patient management. The replies from the free version of ChatGPT were in a natural and positive tone, and the text generated was understandable for college graduates. These findings suggest the potential of LLMs like ChatGPT in assisting individuals with lifestyle-related health concerns and serving as a valuable resource in healthcare settings. Further research and refinement are needed to optimize accuracy and ensure alignment with medical guidelines, promoting safe and effective use in healthcare contexts.

## References

[1] Islam SM, Purnat TD, Phuong NT, Mwingira U, Schacht K, Fröschl G (2014). Non-communicable diseases (NCDs) in developing countries: a symposium report. Global Health.

[2] Ng R, Sutradhar R, Yao Z, Wodchis WP, Rosella LC (2020). Smoking, drinking, diet and physical activity-modifiable lifestyle risk factors and their associations with age to first chronic disease. Int J Epidemiol.

[3] Sharma M, Majumdar PK (2009). Occupational lifestyle diseases: an emerging issue. Indian J Occup Environ Med.

[4] Taber JM, Leyva B, Persoskie A (2015). Why do people avoid medical care? A qualitative study using national data. J Gen Intern Med.

[5] Fallis D, Frické M (2002). Indicators of accuracy of consumer health information on the Internet: a study of indicators relating to information for managing fever in children in the home. J Am Med Inform Assoc.

[6] Sinha RK, Deb Roy A, Kumar N, Mondal H (2023). Applicability of ChatGPT in assisting to solve higher order problems in pathology. Cureus.

[7] Liu J, Wang C, Liu S (2023). Utility of ChatGPT in clinical practice. J Med Internet Res.

[8] Walker HL, Ghani S, Kuemmerli C, Nebiker CA, Müller BP, Raptis DA, Staubli SM (2023). Reliability of medical information provided by ChatGPT: assessment against clinical guidelines and patient information quality instrument. J Med Internet Res.

[9] Dave T, Athaluri SA, Singh S (2023). ChatGPT in medicine: an overview of its applications, advantages, limitations, future prospects, and ethical considerations. Front Artif Intell.

[10] Denecke K, Reichenpfader D (2023). Sentiment analysis of clinical narratives: a scoping review. J Biomed Inform.

[11] Jindal P, MacDermid JC (2017). Assessing reading levels of health information: uses and limitations of flesch formula. Educ Health (Abingdon).

[12] Haupt CE, Marks M (2023). AI-generated medical advice-GPT and beyond. J Am Med Assoc.

[13] Zhavoronkov A (2023). Caution with AI-generated content in biomedicine. Nat Med.

[14] Li H, Moon JT, Iyer D (2023). Decoding radiology reports: potential application of OpenAI ChatGPT to enhance patient understanding of diagnostic reports. Clin Imaging.

[15] Park J, Saha S, Chee B, Taylor J, Beach MC (2021). Physician use of stigmatizing language in patient medical records. JAMA Netw Open.

